# Point Deletion or Insertion in CmeR-Box, A2075G Substitution in 23S rRNA, and Presence of *erm*(B) Are Key Factors of Erythromycin Resistance in *Campylobacter jejuni* and *Campylobacter coli* Isolated From Central China

**DOI:** 10.3389/fmicb.2020.00203

**Published:** 2020-03-03

**Authors:** Yiluo Cheng, Wenting Zhang, Qin Lu, Guoyuan Wen, Zhongzheng Zhao, Qingping Luo, Huabin Shao, Tengfei Zhang

**Affiliations:** Key Laboratory of Prevention and Control Agents for Animal Bacteriosis, Institute of Animal Husbandry and Veterinary, Hubei Academy of Agricultural Sciences, Wuhan, China

**Keywords:** *Campylobacter*, erythromycin resistance, 23S rRNA, *erm*(B), CmeR-Box

## Abstract

*Campylobacter jejuni* and *Campylobacter coli* are major food-borne pathogens that cause bacterial gastroenteritis in humans, and poultry is considered as their most important reservoir. Macrolides, such as erythromycin, are the first-line choice for treatment of campylobacteriosis. In this study, of the 143 *Campylobacter* isolates recovered from poultry in central China during 2015–2017, 25.2% were erythromycin resistant. A2075G substitution in 23S ribosomal RNA (rRNA) and ribosomal methylase encoded by *erm*(B) were found in 4.2 and 4.9% isolates, respectively, and correlated with erythromycin resistance. The polymorphisms of CmeR-Box were also analyzed in our isolates. Among them, 9.1% isolates harbored a point deletion or insertion within the CmeR-Box, and we first showed that point deletion or insertion, but not substitution, in CmeR-Box led to high expression of *cmeABC*, which was significantly associated with erythromycin resistance (*p* < 0.05). These results suggest that point deletion or insertion in CmeR-Box, A2075G substitution in 23S rRNA, and presence of *erm*(B) are three main factors to erythromycin resistance in *C. jejuni* and *C. coli*.

## Introduction

*Campylobacter* species, especially *Campylobacter jejuni* and *Campylobacter coli*, are major food-borne pathogens that cause bacterial gastroenteritis in humans (Huang et al., 2009; Kaakoush et al., 2015). *Campylobacter* is widespread in animals, with poultry as the most important reservoir (Kaakoush et al., 2015). Contaminated poultry products are recognized as the main source of human infection (Zhang T. et al., 2016). In view of the high incidence of fluoroquinolone resistance, including in China (Zhang et al., 2017b), macrolides, such as erythromycin, are the first-line choice for treatment of campylobacteriosis (Bolinger and Kathariou, 2017). Although erythromycin has been limited for use in animal production in China in 2000, the incidence of erythromycin resistance in *Campylobacter* continues to increase (Zhang A. et al., 2016; Du et al., 2018). Therefore, the surveillance of erythromycin-resistant *Campylobacter* is important not only for animal breeding but also for public health.

Macrolides are antibiotics that act by binding to the bacterial 50S ribosomal subunit to obstruct the ribosomal exit tunnel, resulting in inhibition of protein synthesis in bacteria (Dinos, 2017). The A2075G and A2074C/G substitutions in the 23S ribosomal RNA (rRNA) are the most common mechanism for erythromycin resistance in *Campylobacter* (Hao et al., 2009; Perez-Boto et al., 2010; Zhao et al., 2016). Recently, the *erm*(B) gene, which encodes a horizontally transferable ribosomal methylase, was identified in *Campylobacter* (Qin et al., 2014). *erm*(B) can dimethylate a single adenine in the 23S rRNA, leading to the inhibition of the binding of macrolides to the 50S subunit of bacterial ribosomes (Qin et al., 2014; Wang et al., 2014). In addition, various native efflux pumps are encoded in bacteria, which provide baseline resistance levels (Trastoy et al., 2018). *cmeABC* is an important efflux system in *C. jejuni* and *C. coli* (Lin et al., 2002), and the inactivation of *cmeB* results in a significant decrease in the minimum inhibitory concentrations (MICs) of various antibiotics (Ge et al., 2005). CmeR acts as transcriptional repressor by binding to the promoter of *cmeABC* operon to control its expression (Lin et al., 2005a). Mutations in the regulatory region of *cmeABC* promoter (CmeR-Box) have been reported to confer fluoroquinolone resistance in *Campylobacter* (Zhang et al., 2017a; Du et al., 2018), but the effect of these mutations on macrolide resistance has not been investigated.

In this study, to uncover the prevalence and the underlying molecular basis of erythromycin-resistant *Campylobacter* in central China, resistance analysis was conducted, and the mutations on macrolide targets and the present of *erm*(B) were screened. In addition, the polymorphisms of CmeR-Box in the promoter of the *cmeABC* efflux pump were also investigated.

## Materials and Methods

### Ethics Statement

All animal studies were conducted in strict accordance with the animal welfare guidelines of the World Organization for Animal Health. The protocols were approved by the Hubei Provincial Animal Care and Use Committee (approval number SCXK 2015-0021).

### Bacterial Isolation

From 2015 to 2017, 143 *Campylobacter* isolates were collected from chickens or chicken meats in central China (three farms and four markets in Hubei, two farms and three markets in Henan, two farms and two markets in Jiangxi, one farm and two markets in Anhui, and one farm in Hunan), and all the chickens were from commercial broiler flocks. In brief, freshly collected anal and meat swabs were kept into Cary–Blair modified transport media (AMRESCO, United States) and transported to the laboratory for *Campylobacter* isolation. The samples were resuspended in phosphate-buffered saline (PBS) first, and then inoculated in Bolton broth containing *Campylobacter* growth supplement (Oxoid, United Kingdom) and *Campylobacter* Bolton broth selective supplement (Oxoid, United Kingdom) for 24 h at 42°C under microaerobic condition. After inoculation, 100 μl of the culture was spread onto a modified charcoal cefoperazone deoxycholate agar (mCCDA, Oxoid) plate containing *Campylobacter* CCDA-selective supplement (Zhang A. et al., 2016). The suspected *Campylobacter* colonies were identified by polymerase chain reaction (PCR) targeting 16S ribosomal DNA (rDNA) and sequencing as described (Weisburg et al., 1991), and the primers were as follows: 27F, 5′-AGAGTTTGATCMTGGCTCAG-3′; 1492R, 5′-TACGGYTACCTTGTTACGACTT-3′. *C. jejuni* and *C. coli* were differentiated by hippuric acid hydrolysis test and PCR test targeting *C. jejuni*-specific *hipO* gene and *C. coli*-specific *asp* gene (Persson and Olsen, 2005; Keller and Shriver, 2014). To generate a microaerobic environment, all the bacterial culture processes were carried out at 42°C in air tight jars containing the AnaeroPack (Mitsubishi, Japan).

### Antimicrobial Susceptibility Test

According to the European Committee on Antimicrobial Susceptibility Testing guidelines (EUCAST, 2018), erythromycin resistance was first determined by the disk diffusion method on Mueller–Hinton agar (Oxoid, United Kingdom) using erythromycin disks with 15 μg. After incubation for 40 h at 42°C, the diameters (in mm) of the inhibition zones were measured, and <20 mm (*C. jejuni*) or <24 mm (*C. coli*) was determined to be resistant. Then, the MICs of erythromycin-resistant strains were measured by the broth dilution method. In brief, twofold serial dilutions of erythromycin was used at the concentrations of 2–2,048 μg/ml, and 5 × 10^5^ CFU/ml of each isolate was incubated in Mueller–Hinton broth containing serial dilutions of erythromycin under microaerobic condition at 42°C for 24 h. The MICs were determined as the lowest concentration of the agent that completely inhibits visible growth. The antibiotic disks and powder were purchased from Oxoid, United Kingdom. The *Escherichia coli* ATCC 25922 was used as a quality control strain.

### MLST

Multilocus sequence typing (MLST) was carried out in all erythromycin-resistant isolates. In brief, genomic DNA of the *Campylobacter* isolates was extracted using MiniBEST Universal Genomic DNA Extraction Kit (TaKaRa, Dalian, China) according to the manufacturer’s instructions. MLST analysis was conducted by sequencing seven *Campylobacter* housekeeping genes (*aspA*, *glnA*, *gltA*, *glyA*, *pgm*, *tkt*, and *uncA*) as previously described (Dingle et al., 2001). Allele numbers, sequence types (STs) and clonal complexes (CCs) were assigned using the *Campylobacter* MLST database^1^. The calculated tree of the erythromycin-resistant isolates was constructed using the SliptsTree 4 version 1.2 based on the ST types.

### Detection of *erm*(B) and Mutations in 23S rRNA and CmeR-Box

The presence of *erm*(B) was screened by PCR as previously reported (Wang et al., 2014), and the primers used were as follows: ermB-F, 5′-GGGCATTTAACGACGAAACTGG-3′; ermB-R, 5′-CTGTGGTATGGCGGGTAAGT-3′. Polymorphisms present on the amplified fragment of the 23S rRNA and on the promoter of *cmeABC* operon (CmeR-Box) were investigated using PCR and double-stranded DNA sequencing as previously described (Corcoran et al., 2005; Perez-Boto et al., 2010). The primers for amplifying the fragment of the 23S rRNA and CmeR-Box were as follows: 23S-F, 5′-GCTCGAAGGTTAATTGATG-3′ and 23S-R, 5′-GCTCTTGGCAGAACAAC-3′; Cbox1-F, 5′-GG TTGTTACAGGTTGAGGC-3′ and Cbox1-R, 5′-AGCTTAC GCAAAGGATAATG-3′ for *C. jejuni*; and Cbox2-F, 5′-GGTT GTTACAGGTTGAGGC-3′ and Cbox2-R, 5′-AGCTTACGCAAAGGATAATG-3′ for *C. coli*.

### Electrophoresis Mobility Shift Assay

Binding of recombinant CmeR protein to the promoters of the *cmeABC* operon, which contained different CmeR-box sequences, was performed by electrophoresis mobility shift assay (EMSAs) as previously described (Zhang et al., 2012; Grinnage-Pulley and Zhang, 2015). Briefly, the DNA fragments containing CmeR-Box sequences were amplified from genomic DNA of *Campylobacter* of the *Campylobacter* isolates. To obtain the CmeR protein, the coding sequence of *cmeR* was amplified and cloned into vector pET-28a (Novagen, Shanghai, China). *Escherichia coli* BL21(DE3) was transformed with the recombinant plasmid pET28a-*cmeR*, and then, the expression was induced by the addition of 1 mM isopropyl β-D-1-thiogalactopyranoside (IPTG) at 18°C for 12 h. The recombinant CmeR protein was purified with Ni–NTA agarose (Bio-Rad, Shanghai, China) under native conditions, according to the manufacturer’s instructions. Binding reactions were carried out in a 20 μl volume containing 0.1 μg promoter DNA and different amounts of purified recombinant CmeR protein (0, 2, 4, and 8 μg), and incubated at room temperature for 30 min. Electrophoresis was carried out with 5% native polyacrylamide gels at 100 V for 1 h. The gels were stained with 1 μg/ml of ethidium bromide. To compare the bonding abilities, the optical densities of bound DNA and free DNA were measured using Clinx Image Analysis software (Clinx Science Instruments Co., Ltd., Shanghai, China). The proportions of bound DNA were calculated according to the optical densities values (0% was “−”; >0 to ≤50% was “+”; >50 to ≤95% was “++”; >95% was “+++”).

### Real-Time RT-PCR

To analyze the effect of CmeR-Box polymorphism on the expression of *cmeABC*, five *C. jejuni* isolates (QCJ3, JSJ27, XZJ48, WHJ54, and XTJ10) were chosen. The QCJ3 strain contained a wild-type CmeR-Box, the JSJ27 strain contained a point substitution in CmeR-Box, the XZJ48 (ST-7510) and WHJ54 (ST-7512) strains contained the same point deletion in CmeR-Box, and the XTJ10 (ST-7508) strain contained a point insertion in CmeR-Box. None of the isolates contain the *erm*(B) and/or A2075G substitution in the 23S rRNA. Total RNAs of the selected *Campylobacter* isolates were isolated as follows: overnight cultured bacteria were diluted 1:100 in fresh Mueller–Hinton broth and then incubated to mid-log phase (OD_600_ = 0.5) at 42°C under microaerobic condition. Eight micrograms per milliliter (two times of MIC breakpoint by EUCAST, and the breakpoint of erythromycin is 4 μg/ml) of erythromycin was added, and the bacteria were collected at 0, 5, and 120 min post-treatment. Total RNA was isolated and purified using the RNeasy Mini Kit (QIAGEN, Hilden, Germany) according to the manufacturer’s instructions. Then, the isolated RNA was reverse transcribed to complementary DNA (cDNA), and the expression levels of the *cmeA* gene were assessed by real-time reverse transcription PCR (RT-PCR) using SYBRGreen detection (TAKARA BIO INC., Dalian, China) in an ABI7500 system (Thermo Fisher Scientific, CA, United States). The primers for *cmeA* were as follows: qcmeA-F, 5′-CTGACAAGTTTAGCAGGGTA-3′, qcmeA-R, 5′-GCAGCAAAGAAGAAGCACCA-3′. The 16S rRNA and *gapdh* genes were used as the internal control. The primers were as follows: q16S-F, 5′-TACCTGGGCTTGATATCCTA-3′, q16S-R, 5′-GGACTTAACCCAACATCTCA-3′; qgapdh-F, 5′-AGGCAGTGTTGATAGTGAAGG-3′, qgapdh-R, 5′-CAATTTGTGCGCCGTGTT-3′. The expression level of *cmeA* before treatment of the strain harboring wild-type CmeR-Box (*C. jejuni* strain QCJ3) was used as control condition. Each assay was carried out with at least three biological replicates. Differences in relative transcript abundance level were calculated using the 2^–ΔΔCT^ method (Pfaffl, 2001), and ≥2-fold changes were considered as differentially expressed.

### Statistical Analysis

The Fisher test was used to identify the correlation between resistance and mutations, and the Student’s *t*-test was used to compare the expression levels of target genes of different strains. The analysis was carried out by SPSS 19.0. A probability (*p*) value of <0.05 was considered statistically significant.

## Results

### Prevalence of Erythromycin-Resistant *Campylobacter*

**FIGURE 1:**
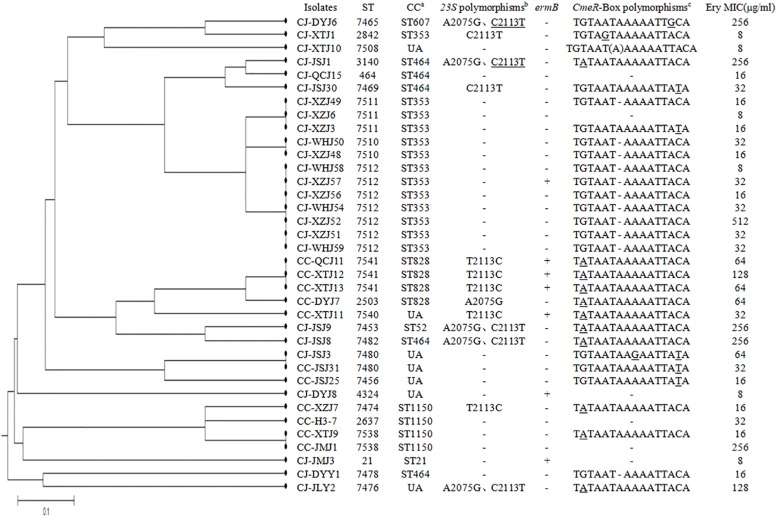
Dendrogram of multilocus sequence typing (MLST) profiles among the 36 erythromycin-resistant *Campylobacter* isolates. Corresponding minimum inhibitory concentrations (MICs) of erythromycin, erythromycin-resistant genes, mutations found, and the CmeR-Box sequence are listed for each isolate. ^a^UA means unallocated in campylobacters. ^b^The underline indicates that two of the three copies were mutated. ^c^Underline means point substitution, “(A)” means point insertion, “–” means point deletion.

A total of 143 *Campylobacter* strains, including 83 *C. jejuni* and 60 *C. coli* isolates, were isolated and tested for erythromycin susceptibility. Among them, 36 (25.2%) *Campylobacter* isolates were erythromycin resistant, including 25 (30.1%, MIC > 4 mg/l) *C. jejuni* and 11 (18.3%, MIC > 8 mg/l) *C. coli* (Figure 1). These results showed high erythromycin resistance rates of *C. jejuni* and *C. coli* isolates in our study.

### Mutations in 23S rRNA of *Campylobacter* Isolates

Polymorphisms on the 23S rRNA were analyzed in all *Campylobacter* isolates. As shown in Table 1, Figure 1, and Supplementary Material, point substitutions on 23S rRNA were found in 23 isolates (8 *C. jejuni* and 15 *C. coli* isolates). According to published sequences, the *Campylobacter* genome encodes three copies of 23S rRNA (Sheppard and Maiden, 2015). Except for the isolates that contained A2075G substitution, 16 isolates contained the C2113T (*C. jejuni*) or T2113C (*C. coli*) substitution in all three copies of 23S rRNA, while one *C. jejuni* contained this substitution in two of three copies (double peak and the peak for guanine was two times higher than the peak for adenine), and among them, 7 of the 17 isolates were erythromycin resistant; thus, the correlation was not significant (*p* > 0.05). The A2075G substitution was observed in 6 (5 *C. jejuni* and 1 *C. coli* isolates) of the 23 isolates, and all of them were erythromycin resistant. These results suggested that the A2075G, but not the C2113T or T2113C, substitution in 23S rRNA was responsible for erythromycin resistance in *Campylobacter*.

**TABLE 1:** Polymorphisms on the 23S rRNA gene of *C. jejuni* and *C. coli* isolates.

	Position^c^	Number of isolates	Number of resistant isolates	Resistance frequency (%)
***C. jejuni***				
Mutations in 23S rRNA^a^	A2075G(3/3), C2113T(2/3)	2	2	100
	A2075G(3/3), C2113T(3/3)	3	3	100
	C2113T(2/3)	1	0	0
	C2113T(3/3)	2	2	100
Without mutation	–	75	18	24.0
***C. coli***				
Mutations in 23S rRNA^b^	A2075G(3/3)	1	1	100
	T2113C(3/3)	14	5	35.7
Without mutation	–	45	5	11.1

*^*a*^The mutations were determined based on the sequence of the reference strain *C. jejuni* NCTC 11168 (accession number: AL111168.1). ^*b*^The mutations were determined based on the sequence of the reference strain *C. coli* OR12 (accession number: CP013733.1) (O’Kane and Connerton, 2017). ^*c*^The numbers in parentheses represent the number of mutations in the three copies of the 23S rRNA gene.*

### Presence of *erm*(B) in *Campylobacter* Isolates

The ribosomal methylase *erm*(B) gene was found in seven *Campylobacter* isolates (4.9%), including three *C. jejuni* and four *C. coli*. These seven isolates were also erythromycin resistant, and none of them contained an A2075G substitution in 23S rRNA (Figure 1; Wang et al., 2014). Although the incidence of *erm*(B) was not high, the seven *erm*(B)-harboring strains were isolated from five different regions.

### Polymorphism Analysis of CmeR-Box in *Campylobacter* Isolates

**FIGURE 2:**
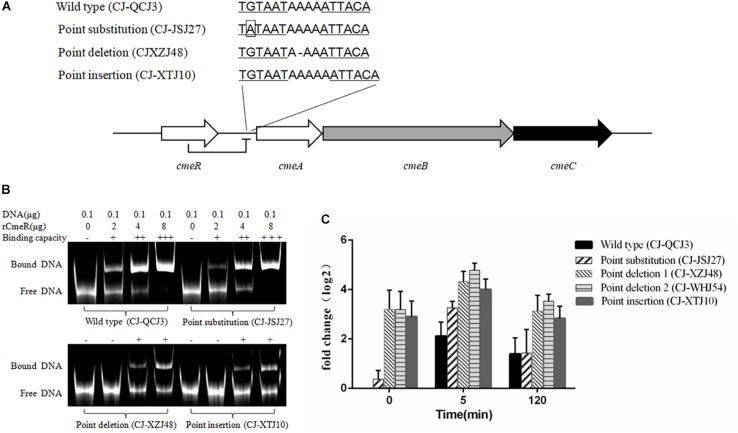
*cmeA* expression levels in *Campylobacter* isolates with different CmeR-Box sequences. **(A)** CmeR-Box sequences in the *Campylobacter* isolates. **(B)** Binding of CmeR to the variant *cmeABC* promoter DNA. **(C)** The expression levels of *cmeA* gene at 5- and 120-min post-erythromycin treatment in the *Campylobacter* isolates harboring different CmeR-Box sequences. Each assay was carried out at least in three biological replicates, and the bars mean the standard deviation in each assay.

Polymorphisms of CmeR-Box were analyzed in all isolates, and seven CmeR-Box variants were identified (Figure 1 and Table 2). Among them, 13 (9.1%) isolates contained a point deletion/insertion within the inverted sequences (Figure 2A), and 12 out of 13 were resistant to erythromycin. Among the 12 resistant isolates, 11 did not contain *erm*(B) or a mutation on 23S rRNA. Statistical analysis showed that the occurrence of point deletion/insertions significantly correlated with erythromycin resistance (*p* < 0.05), suggesting an important role in the erythromycin resistance phenotype. In contrast, point substitutions in CmeR-Box were found in 41 (49.4%) *C. jejuni* isolates and 36 (60.0%) *C. coli* isolates, but among them, only 9 (22.0%) *C. jejuni* and 9 (25.0%) *C. coli* isolates were erythromycin resistant, and among the 18 resistant isolates, most of them also harbor mutations in 23S rRNA and/or the *erm*(B) gene (Figure 1). Statistical analysis showed that nucleotide substitution was not correlated with erythromycin resistance (*p* > 0.05). Our results suggested that point deletions or insertions, but not nucleotide substitution, in the CmeR-Box was significantly associated with erythromycin resistance.

**TABLE 2:** CmeR-Box polymorphisms in *C. jejuni* and *C. coli* isolates.

	Number of isolates	Number of resistant isolates^a^	Resistance frequency (%)	vs. None^b^
***C. jejuni***				
Point substitution in CmeR-Box	41	9(5)	22.0	*p* > 0.05
Point deletion or insertion in CmeR-Box	13	12(1)	92.3	*p* < 0.01
Without mutation	29	4(2)	13.8	–
***C. coli***				
Point substitution in CmeR-Box	36	9(5)	25.0	*p* > 0.05
Without mutation	24	2(0)	8.3	–

*^*a*^The numbers in brackets indicate the strains containing A2075G substitution and/or *erm*(B). ^*b*^The correlation between resistance and mutations were calculated with the Fisher test.*

### EMSAs and Expression Analysis of *cmeABC*

To further understand the effect of point deletion/insertion in the CmeR-Box on erythromycin resistance, the binding of CmeR to the CmeR-Box with a point deletion/insertion was confirmed with the EMSAs. As shown in Figure 2B, the binding of CmeR to CmeR-Box with a point deletion/insertion was significantly decreased, suggesting that the *cmeABC* operon in these isolates was highly derepressed and associated with erythromycin resistance. Analysis of the expression levels of *cmeA* at the time point 0 min further confirmed these results (Figure 2C). Although *cmeA* expression could be induced by the addition of erythromycin in all the isolates, as compared with the isolate without a mutation in the CmeR-Box, higher expression was observed in the isolates harboring a point deletion/insertion before and after erythromycin treatment (*p* < 0.05). It is worth noting that, in the isolates harboring a point deletion/insertion, *cmeA* expression was originally high before erythromycin treatment and only increased by approximately twofold at 5 min post-treatment and recovered at 2 h post-treatment. In contrast, in the wild-type isolate and isolate with a point substitution, although *cmeA* expression was upregulated by more than fourfold at 5 min post-treatment, their expression levels were still lower than in isolates with a point deletion/insertion.

### Genetic Diversity Analysis

The genetic diversity of erythromycin-resistant *Campylobacter* isolates was analyzed with MLST, as previously described (Dingle et al., 2001). As shown in Figure 1, the 36 erythromycin-resistant *Campylobacter* isolates were distributed along 23 STs and 8 CCs. The dominant CC was CC353, and seven isolates could not be assigned to a CC. Five *C. jejuni* strains with an A2075G substitution in the 23S rRNA belonged to three CCs (CC607, CC464, and CC52), and one could not be assigned to a CC. Three *C. jejuni erm*(B)-harboring strains belonged to two different CCs (CC353 and CC21), and one was not assigned. These results suggest a diverse distribution of genotypes in *C. jejuni* resistant to erythromycin. In contrast, 10 out of 11 erythromycin-resistant *C. jejuni* with a point deletion in the CmeR-Box were distributed between two STs both belonging to the dominant CC353, suggesting a common ancestor. In *C. coli* isolates, three *erm*(B)-harboring isolates shared the same CC (CC828) clustered with an unallocated (UA) strain, suggesting a common ancestor.

## Discussion

*Campylobacter jejuni* and *Campylobacter coli* are major food-borne pathogens worldwide, and poultry is recognized as the most important reservoir of these pathogens (Crawshaw, 2019; Thomas et al., 2019). Usually, erythromycin is the first-line choice for treatment of campylobacteriosis (Bolinger and Kathariou, 2017). Therefore, to investigate the prevalence and erythromycin resistance mechanisms of *C. jejuni* and *C. coli* is not only important for the poultry industry but also for public health.

In our study, 30.1% *C. jejuni* and 18.3% *C. coli* isolates were erythromycin resistant. As previously reported, the prevalence of macrolide resistance is common in *C. coli* but remains low in *C. jejuni*. For example, data from the National Antimicrobial Resistance Monitoring System (NARMS) report of the U.S. Food and Drug Administration on human clinical isolates and food-producing animal isolates indicated that the resistance rate in *C. jejuni* was under 4% in 2014 (FDA, 2014). In a previous investigation in China, the global resistance rate of *Campylobacter* to erythromycin was 18.4%, and among the resistant strains, 97.5% were *C. coli* and 2.5% were *C. jejuni* (Zhang A. et al., 2016). However, in this study, high erythromycin resistance rates were found in both *C. jejuni* and *C. coli* isolated from central China, which pressed us to further understand the molecular mechanisms of erythromycin resistance in these isolates.

Mutations in 23S rRNA, and single adenine methylation in the 23S rRNA by ribosomal methylase *erm*(B), are two well-known mechanisms for erythromycin resistance in *Campylobacter* (Zhao et al., 2016). In this study, A2075G substitution in 23S rRNA and ribosomal methylase encoded by *erm*(B) were found in 4.2 and 4.9% isolates, respectively, and all of them were erythromycin resistant. As previously reported, the A2075G substitution is one of the most prevalent genetic events conferring high-level resistance to erythromycin (Zhao et al., 2016), as further evidenced by our results. Substitutions at 2113 site were found in our isolates. Based on previous reports and the sequenced reference strains, including *C. jejuni* NCTC 11168 and *C. coli* OR12 (Parkhill et al., 2000; Perez-Boto et al., 2010; O’Kane and Connerton, 2017), the dominant nucleotide at 2113 site is “C” in *C. jejuni* and “T” in *C. coli*, which were defined as wild type in both species in this study. Correlation analysis showed that this substitution was not correlated with erythromycin resistance (*p* > 0.05). We infer that this substitution exists extensively in *Campylobacter* and does not affect erythromycin resistance. Some of the resistant isolates with this substitution could be explained by other resistance factors, such as *erm*(B) in strains QCJ11, XTJ11, XTJ12, and XTJ13. However, the causes of resistance on the part of isolates are still unknown and require further study.

*erm*(B)-harboring *Campylobacter* isolates were first reported in 2014 in China (Qin et al., 2014) and then detected in Spain and the United States (Florez-Cuadrado et al., 2016). In this study, although the incidence of *erm*(B) was not high, seven *erm*(B)-harboring strains were isolated from five different regions. Our results suggested that *erm*(B) might be widespread at the regional distribution in central China.

Drug efflux is another important resistance mechanism conferred by native efflux systems in bacteria. The *cmeABC* operon, which encodes a drug efflux pump and plays an important role in drug resistance in *Campylobacter* (Lin et al., 2005a), is negatively regulated by CmeR by binding to a 16-base inverted repeat sequence [CmeR-Box, TGTAATA(or T)TTTATTACA] in the promoter region (Yan et al., 2006). In our isolates, a couple of CmeR-Box variants were identified (Figure 2A and Table 2), including point substitutions and deletion/insertion within the inverted sequences. It is interesting that point deletions/insertions were found in 13 (9.1%) isolates, and 12 out of 13 isolates were resistant, which was correlated to erythromycin resistance (*p* < 0.05). However, *Campylobacter* harboring point deletions in CmeR-Box were seldom reported and not well characterized (Abd El-Tawab et al., 2019). In contrast, point substitutions were not correlated to erythromycin resistance (*p* > 0.05). Substitutions in CmeR-Box have been reported to cause overexpression of *cmeABC* although not affecting the susceptibility of *C. jejuni* to most tested antimicrobials including erythromycin (Grinnage-Pulley and Zhang, 2015). It is worth noting that all of the mutations identified in CmeR-Box were point substitutions in that report, so their result was consistent with our finding that there was no correlation between point substitutions in CmeR-Box and erythromycin resistance. In our study, point deletion/insertion in CmeR-Box was identified in *C. jejuni* isolates, and our results suggested that point deletion or insertion, but not substitution, in CmeR-Box was significantly associated with erythromycin resistance.

To further investigate the effect of point deletion/insertion in CmeR-Box on resistance, the binding of CmeR to the various CmeR-Box and the expressional levels of *cmeABC* were detected. It is well-known that CmeR can interact with bile salts and transduce this signaling, but little is known about the capacity of antibiotics to induce *cmeABC* expression (Lin et al., 2005b). In this study, the expression of *cmeA* was induced after erythromycin treatment. We found that the expression of *cmeR*, which encodes the repressor of *cmeABC*, was slightly reduced 5 min post-treatment, and there were no significant difference between these tested isolates (data not shown). We inferred that the upregulated expression of *cmeABC* after erythromycin treatment was due to the reduced expression of its repressor CmeR. Comparing with the wild-type CmeR-Box and CmeR-Box with point substitution, an obvious decrease in binding ability of CmeR to CmeR-Box with a point deletion/insertion, and subsequent higher overexpression of *cmeABC*, was showed. These results support why point deletion or insertion, but not substitutions, in CmeR-Box was significantly associated with erythromycin resistance. Functional CmeR is a dimer, and each monomer binds to one half of the inverted repeat of the CmeR-Box (Gu et al., 2007; Lei et al., 2011). We inferred that, although point substitutions reduced the bonding strength of CmeR to CmeR-Box, the bonding strength was still moderate (Figure 2B). Therefore, the expression of *cmeABC* was only slightly increased (Figure 2B) in these isolates harboring CmeR-Box with point substitutions. The slightly increased expression of *cmeABC* might result in increased MICs, but the MICs did not reach the breakpoint in these isolates. In contrast, a point deletion/insertion changed the distance between two consecutive binding sequences, leading to an obvious decrease in binding ability and subsequent overexpression of *cmeABC* (Figure 2), which conferred high level of erythromycin resistance. With regard to that one susceptible isolate carrying point deletion in CmeR-Box, we infer that there might be some unknown mutations, such as the mutations in the *cmeABC* proteins, which affect its resistant ability. CC-JMJ1 is a specific highly resistant isolate, which does not contain any mutations in CmeR-Box and 23S rRNA, and for *erm*(B), we infer that it may contain a previously reported resistance-enhancing variant of the efflux pump *cmeABC*, or due to other unknown mechanisms (Yao et al., 2016).

## Conclusion

In summary, 25.2% of *Campylobacter* isolates in central China were erythromycin resistant. The A2075G substitution in 23S rRNA and the presence of *erm*(B) were identified as two important factors that lead to erythromycin resistance. Furthermore, this is the first study to report that a point deletion/insertion, but not substitution, in CmeR-Box could significantly increase the expression of *cmeABC*, which plays important roles in erythromycin resistance. These findings will help us to further understand the mechanism of erythromycin resistance in *Campylobacter*.

## Data Availability Statement

The raw data supporting the conclusions of this article will be made available by the authors, without undue reservation, to any qualified researcher.

## Ethics Statement

All animal studies were conducted in strict accordance with the animal welfare guidelines of the World Organization for Animal Health. The protocols were approved by the Hubei Provincial Animal Care and Use Committee (approval number SCXK 2015-0021).

## Author Contributions

YC, TZ, and HS conceived and designed the experiments and wrote the manuscript. YC, QL, QLo, and ZZ performed the experiments. GW and WZ analyzed the data.

## Conflict of Interest

The authors declare that the research was conducted in the absence of any commercial or financial relationships that could be construed as a potential conflict of interest.

## Footnotes

1 http://pubmlst.org/campylobacter

## References

[B1] Abd El-Tawab A. A. Ammar A. M. Ahmed H. A. Hefny A. A. (2019). Efflux pump inhibitors, Alpha-Tocopherol and aspirin: role in *Campylobacter jejuni* and *Campylobacter coli* fluoroquinolone resistance. *Microb. Drug Resist.* 25 203–211. 10.1089/mdr.2018.0086 30277840

[B2] Bolinger H. Kathariou S. (2017). The current state of macrolide resistance in *Campylobacter* spp.: trends and impacts of resistance mechanisms. *Appl. Environ. Microbiol.* 83 e416–e417. 10.1128/AEM.00416-17 28411226 PMC5452823

[B3] Corcoran D. Quinn T. Cotter L. O’halloran F. Fanning S. (2005). Characterization of a cmeABC operon in a quinolone-resistant Campylobacter coli isolate of Irish origin. *Microb. Drug Resist.* 11 303–308. 10.1089/mdr.2005.11.303 16359189

[B4] Crawshaw T. (2019). A review of the novel thermophilic *Campylobacter*, *Campylobacter hepaticus*, a pathogen of poultry. *Transbound. Emerg. Dis.* 66 1481–1492. 10.1111/tbed.13229 31081981

[B5] Dingle K. E. Colles F. M. Wareing D. R. Ure R. Fox A. J. Bolton F. E. (2001). Multilocus sequence typing system for *Campylobacter jejuni*. *J. Clin. Microbiol.* 39 14–23. 11136741 10.1128/JCM.39.1.14-23.2001 PMC87672

[B6] Dinos G. P. (2017). The macrolide antibiotic renaissance. *Br. J. Pharmacol.* 174 2967–2983. 10.1111/bph.13936 28664582 PMC5573421

[B7] Du Y. Wang C. Ye Y. Liu Y. Wang A. Li Y. (2018). Molecular identification of multidrug-resistant *Campylobacter* species from Diarrheal patients and poultry meat in Shanghai China. *Front. Microbiol.* 9:1642. 10.3389/fmicb.2018.01642 30108555 PMC6079250

[B8] EUCAST (2018). *Breakpoint Tables for Interpretation of MICs and Zone Diameters. Version 8.1, 2018*. Available online at: http://www.eucast.org

[B9] FDA (2014). “The national antimicrobial resistance monitoring system (NARMS): enteric bacteria,” in *NARMS Integrated Report: 2014*, (Silver Spring, MA: US Food and Drug Administration).

[B10] Florez-Cuadrado D. Ugarte-Ruiz M. Quesada A. Palomo G. Domínguez L. Porrero M. C. (2016). Description of an *erm*(B)-carrying *Campylobacter coli* isolate in Europe. *J. Antimicrob. Chemother.* 71 841–843. 10.1093/jac/dkv383 26604242

[B11] Ge B. Mcdermott P. F. White D. G. Meng J. (2005). Role of efflux pumps and topoisomerase mutations in fluoroquinolone resistance in *Campylobacter jejuni* and *Campylobacter coli*. *Antimicrob. Agents Chemother.* 49 3347–3354. 10.1128/aac.49.8.3347-3354.2005 16048946 PMC1196287

[B12] Grinnage-Pulley T. Zhang Q. (2015). Genetic basis and functional consequences of differential expression of the CmeABC efflux pump in *Campylobacter jejuni* isolates. *PLoS One* 10:e0131534. 10.1371/journal.pone.0131534 26132196 PMC4488513

[B13] Gu R. Su C. C. Shi F. Li M. Mcdermott G. Zhang Q. (2007). Crystal structure of the transcriptional regulator CmeR from *Campylobacter jejuni*. *J. Mol. Biol.* 372 583–593. 10.1016/j.jmb.2007.06.072 17686491 PMC2104645

[B14] Hao H. Dai M. Wang Y. Peng D. Liu Z. Yuan Z. (2009). 23S rRNA mutation A2074C conferring high-level macrolide resistance and fitness cost in *Campylobacter jejuni*. *Microb. Drug Resist.* 15 239–244. 10.1089/mdr.2009.0008 19857128

[B15] Huang J. L. Xu H. Y. Bao G. Y. Zhou X. H. Ji D. J. Zhang G. (2009). Epidemiological surveillance of *Campylobacter jejuni* in chicken, dairy cattle and diarrhoea patients. *Epidemiol. Infect.* 137 1111–1120. 10.1017/S0950268809002039 19192321

[B16] Kaakoush N. O. Castaño-Rodríguez N. Mitchell H. M. Si M. M. (2015). Global epidemiology of *Campylobacter* infection. *Clin. Microbiol. Rev* 28 687–720. 10.1128/CMR.00006-15 26062576 PMC4462680

[B17] Keller J. I. Shriver W. G. (2014). Prevalence of three campylobacter species, *C. jejuni*, *C. coli*, and *C. lari*, using multilocus sequence typing in wild birds of the Mid-Atlantic region, USA. *J. Wildl. Dis.* 50 31–41. 10.7589/2013-06-136 24171567

[B18] Lei H. T. Shen Z. Surana P. Routh M. D. Su C. C. Zhang Q. (2011). Crystal structures of CmeR-bile acid complexes from *Campylobacter jejuni*. *Protein Sci.* 20 712–723. 10.1002/pro.602 21328631 PMC3081549

[B19] Lin J. Akiba M. Sahin O. Zhang Q. (2005a). CmeR functions as a transcriptional repressor for the multidrug efflux pump CmeABC in *Campylobacter jejuni*. *Antimicrob. Agents Chemother.* 49 1067–1075. 10.1128/aac.49.3.1067-1075.2005 15728904 PMC549222

[B20] Lin J. Cagliero C. Guo B. Barton Y. W. Maurel M. C. Payot S. (2005b). Bile salts modulate expression of the CmeABC multidrug efflux pump in *Campylobacter jejuni*. *J. Bacteriol.* 187 7417–7424. 10.1128/jb.187.21.7417-7424.2005 16237025 PMC1272998

[B21] Lin J. Michel L. O. Zhang Q. (2002). CmeABC functions as a multidrug efflux system in *Campylobacter jejuni*. *Antimicrob. Agents Chemother.* 46 2124–2131. 10.1128/aac.46.7.2124-2131.2002 12069964 PMC127319

[B22] O’Kane P. M. Connerton I. F. (2017). Characterisation of aerotolerant forms of a robust chicken colonizing *Campylobacter coli*. *Front. Microbiol.* 8:513. 10.3389/fmicb.2017.00513 28396658 PMC5366326

[B23] Parkhill J. Wren B. W. Mungall K. Ketley J. M. Churcher C. Basham D. (2000). The genome sequence of the food-borne pathogen Campylobacter jejuni reveals hypervariable sequences. *Nature* 403 665–668. 10.1038/35001088 10688204

[B24] Perez-Boto D. Lopez-Portoles J. A. Simon C. Valdezate S. Echeita M. A. (2010). Study of the molecular mechanisms involved in high-level macrolide resistance of Spanish *Campylobacter jejuni* and *Campylobacter coli* strains. *J. Antimicrob. Chemother.* 65 2083–2088. 10.1093/jac/dkq268 20647243

[B25] Persson S. Olsen K. E. (2005). Multiplex PCR for identification of *Campylobacter coli* and *Campylobacter jejuni* from pure cultures and directly on stool samples. *J. Med. Microbiol.* 54 1043–1047. 10.1099/jmm.0.46203-0 16192435

[B26] Pfaffl M. W. (2001). A new mathematical model for relative quantification in real-time RT-PCR. *Nucleic Acids Res.* 29:e45. 11328886 10.1093/nar/29.9.e45 PMC55695

[B27] Qin S. Wang Y. Zhang Q. Zhang M. Deng F. Shen Z. (2014). Report of ribosomal RNA methylase gene erm(B) in multidrug-resistant *Campylobacter coli*. *J. Antimicrob. Chemother.* 69 964–968. 10.1093/jac/dkt492 24335515

[B28] Sheppard S. K. Maiden M. C. (2015). The evolution of *Campylobacter jejuni* and *Campylobacter coli*. *Cold Spring Harb. Perspect. Biol.* 7:a018119. 10.1101/cshperspect.a018119 26101080 PMC4526750

[B29] Thomas K. M. de Glanville W. A. Barker G. C. Benschop J. Buza J. J. Cleaveland S. (2019). Prevalence of *Campylobacter* and *Salmonella* in African food animals and meat: a systematic review and meta-analysis. *Int. J. Food Microbiol.* 315:108382. 10.1016/j.ijfoodmicro.2019.108382 31710971 PMC6985902

[B30] Trastoy R. Manso T. Fernandez-Garcia L. Blasco L. Ambroa A. Perez Del Molino M. L. (2018). Mechanisms of bacterial tolerance and persistence in the gastrointestinal and respiratory environments. *Clin. Microbiol. Rev.* 31 e23–e18. 10.1128/CMR.00023-18 30068737 PMC6148185

[B31] Wang Y. Zhang M. Deng F. Shen Z. Wu C. Zhang J. (2014). Emergence of multidrug-resistant *Campylobacter* species isolates with a horizontally acquired rRNAmethylase. *Antimicrob. Agents Chemother.* 58 5405–5412. 10.1128/AAC.03039-14 24982085 PMC4135855

[B32] Weisburg W. G. Barns S. M. Pelletier D. A. Lane D. J. (1991). 0.16S ribosomal DNA amplification for phylogenetic study. *J. Bacteriol.* 173 697–703. 10.1128/jb.173.2.697-703.1991 1987160 PMC207061

[B33] Yan M. Sahin O. Lin J. Zhang Q. (2006). Role of the CmeABC efflux pump in the emergence of fluoroquinolone-resistant *Campylobacter* under selection pressure. *J. Antimicrob. Chemother.* 58 1154–1159. 10.1093/jac/dkl412 17023497

[B34] Yao H. Shen Z. Wang Y. Deng F. Liu D. Naren G. (2016). Emergence of a potent multidrug efflux pump variant that enhances *Campylobacter* resistance to multiple antibiotics. *mBio* 7:e001543-16. 10.1128/mBio.01543-16 27651364 PMC5030363

[B35] Zhang A. Song L. Liang H. Gu Y. Zhang C. Liu X. (2016). Molecular subtyping and erythromycin resistance of *Campylobacter* in China. *J. Appl. Microbiol.* 121 287–293. 10.1111/jam.13135 26999516

[B37] Zhang T. Cheng Y. Luo Q. Lu Q. Dong J. Zhang R. (2017a). Correlation between gyrA and CmeR box polymorphism and fluoroquinolone resistance in *Campylobacter jejuni* isolates in China. *Antimicrob. Agents Chemother.* 61:e00422-17. 10.1128/AAC.00422-17 28438942 PMC5487682

[B38] Zhang T. Ding Y. Li T. Wan Y. Li W. Chen H. (2012). A Fur-like protein PerR regulates two oxidative stress response related operons dpr and metQIN in *Streptococcus suis*. *BMC Microbiol.* 12:85. 10.1186/1471-2180-12-85 22646062 PMC3458967

[B39] Zhang T. Dong J. Cheng Y. Lu Q. Luo Q. Wen G. (2017b). Genotypic diversity, antimicrobial resistance and biofilm-forming abilities of *Campylobacter* isolated from chicken in Central China. *Gut Pathog.* 9 62. 10.1186/s13099-017-0209-6 29151896 PMC5680748

[B36] Zhang T. Luo Q. Chen Y. Li T. Wen G. Zhang R. (2016). Molecular epidemiology, virulence determinants and antimicrobial resistance of *Campylobacter* spreading in retail chicken meat in Central China. *Gut Pathog.* 8:48. 27800028 10.1186/s13099-016-0132-2 PMC5080698

[B40] Zhao S. Tyson G. H. Chen Y. Li C. Mukherjee S. Young S. (2016). Whole-genome sequencing analysis accurately predicts antimicrobial resistance phenotypes in *Campylobacter* spp. *Appl Environ Microbiol* 82 459–466. 10.1128/AEM.02873-15 26519386 PMC4711122

