# Supplementary material for: Point Deletion or Insertion in CmeR-Box, A2075G Substitution in 23S rRNA, and Presence of erm(B) Are Key Factors of Erythromycin Resistance in Campylobacter jejuni and Campylobacter coli Isolated From Central China
Source: Front Microbiol. 2020 Mar 3;11:203. doi: 10.3389/fmicb.2020.00203 (PMC7062675; doi:10.3389/fmicb.2020.00203)
Supplement: Supplementary file 1 [file Table_1.DOCX]

**Supplemental material 1:** the sequences of the 23S rRNA in this study.

The nucleotide marked with green indicated the wild-type nucleotide at points 2075 and 2113; the nucleotide marked with red indicated the mutation at points 2075 or 2113 in all of the three copies of the 23S rRNA; the nucleotide marked with yellow indicated the mutation at point 2113 in two of the three copies of the 23S rRNA

1. Wild-type 23S rRNA of *Campylobacter jejuni*:

23S rRNA *o*f *C. jejuni* reference strain NCTC 11168 (Accession number: AL111168.1). GCTCGAAGGTTAATTGATGGGGTTAGCATTAGCGAAGCTCTTGATCGAAGCCCGAGTAAACGGCGGCCGTAACTATAACGGTCCTAAGGTAGCGAAATTCCTTGTCGGTTAAATACCGACCTGCATGAATGGCGTAACGAGATGGGAGCTGTCTCAAAGAGGGATCCAGTGAAATTGTAGTGGAGGTGAAAATTCCTCCTACCCGCGGCAAGACGGAAAGACCCCGTGGACCTTTACTACAGCTTGACACTGCTACTTGGATAAGAATGTGCAGGATAGGTGGGAGGCTTTGAGTATATGACGCCAGTTGTATATGAGCCATTGTTGAGATACCACTCTTTCTTATTTGGGTAGCTAACCAGCTTGAGTTATCCTCAAGTGGGACAATGTCTGGTGGGTAGTTTGACTGGGGCGGTCGCCTCCCAAATAATAACGGAGGCTTACAAAGGTTGGCTCAGAACGGTTGGAAATCGTTCGTAGAGTATAAAGGTATAAGCCAGCTTAACTGCAAGACATACAAGTCAAGCAGAGACGAAAGTCGGTCTTAGTGATCCGGTGGTTCTGTGTGGAAGGGCCATCGCTCAAAGGATAAAAGGTACCCCGGGGATAACAGGCTGATCTCCCCCAAGAGCTCACATCGACGGGGAGGTTTGGCACCTCGATGTCGGCTCATCGCATCCTGGGGCTGGAGCAGGTCCCAAGGGTATGGCTGTTCGCCATTTAAAGCGGTACGCGAGCTGGGTTCAGAACGTCGTGAGACAGTTCGGTCCCTATCTGCCGTGGGCGTAAGAAGATTGAAGAGATTTGACCCTAGTACGAGAGGACCGGGTTGAACAAACCACTGGTGTAGCTGTTGTTCTGCCAAGAGC

1. Mutation of 23S rRNA in *C. jejuni*
2. A2075G and C2113T mutations present in all of the three copies of the 23S rRNA

GCTCGAAGGTTAATTGATGGGGTTAGCATTAGCGAAGCTCTTGATCGAAGCCCGAGTAAACGGCGGCCGTAACTATAACGGTCCTAAGGTAGCGAAATTCCTTGTCGGTTAAATACCGACCTGCATGAATGGCGTAACGAGATGGGAGCTGTCTCAAAGAGGGATCCAGTGAAATTGTAGTGGAGGTGAAAATTCCTCCTACCCGCGGCAAGACGGAGAGACCCCGTGGACCTTTACTACAGCTTGACACTGCTATTTGGATAAGAATGTGCAGGATAGGTGGGAGGCTTTGAGTATATGACGCCAGTTGTATATGAGCCATTGTTGAGATACCACTCTTTCTTATTTGGGTAGCTAACCAGCTTGAGTTATCCTCAAGTGGGACAATGTCTGGTGGGTAGTTTGACTGGGGCGGTCGCCTCCCAAATAATAACGGAGGCTTACAAAGGTTGGCTCAGAACGGTTGGAAATCGTTCGTAGAGTATAAAGGTATAAGCCAGCTTAACTGCAAGACATACAAGTCAAGCAGAGACGAAAGTCGGTCTTAGTGATCCGGTGGTTCTGTGTGGAAGGGCCATCGCTCAAAGGATAAAAGGTACCCCGGGGATAACAGGCTGATCTCCCCCAAGAGCTCACATCGACGGGGAGGTTTGGCACCTCGATGTCGGCTCATCGCATCCTGGGGCTGGAGCAGGTCCCAAGGGTATGGCTGTTCGCCATTTAAAGCGGTACGCGAGCTGGGTTCAGAACGTCGTGAGACAGTTCGGTCCCTATCTGCCGTGGGCGTAAGAAGATTGAAGAGATTTGACCCTAGTACGAGAGGACCGGGTTGAACAAACCACTGGTGTAGCTGTTGTTCTGCCAAGAGC

1. C2113T mutations present in two of the three copies and A2075G in 23S rRNA

GCTCGAAGGTTAATTGATGGGGTTAGCATTAGCGAAGCTCTTGATCGAAGCCCGAGTAAACGGCGGCCGTAACTATAACGGTCCTAAGGTAGCGAAATTCCTTGTCGGTTAAATACCGACCTGCATGAATGGCGTAACGAGATGGGAGCTGTCTCAAAGAGGGATCCAGTGAAATTGTAGTGGAGGTGAAAATTCCTCCTACCCGCGGCAAGACGGAGAGACCCCGTGGACCTTTACTACAGCTTGACACTGCTATTTGGATAAGAATGTGCAGGATAGGTGGGAGGCTTTGAGTATATGACGCCAGTTGTATATGAGCCATTGTTGAGATACCACTCTTTCTTATTTGGGTAGCTAACCAGCTTGAGTTATCCTCAAGTGGGACAATGTCTGGTGGGTAGTTTGACTGGGGCGGTCGCCTCCCAAATAATAACGGAGGCTTACAAAGGTTGGCTCAGAACGGTTGGAAATCGTTCGTAGAGTATAAAGGTATAAGCCAGCTTAACTGCAAGACATACAAGTCAAGCAGAGACGAAAGTCGGTCTTAGTGATCCGGTGGTTCTGTGTGGAAGGGCCATCGCTCAAAGGATAAAAGGTACCCCGGGGATAACAGGCTGATCTCCCCCAAGAGCTCACATCGACGGGGAGGTTTGGCACCTCGATGTCGGCTCATCGCATCCTGGGGCTGGAGCAGGTCCCAAGGGTATGGCTGTTCGCCATTTAAAGCGGTACGCGAGCTGGGTTCAGAACGTCGTGAGACAGTTCGGTCCCTATCTGCCGTGGGCGTAAGAAGATTGAAGAGATTTGACCCTAGTACGAGAGGACCGGGTTGAACAAACCACTGGTGTAGCTGTTGTTCTGCCAAGAGC


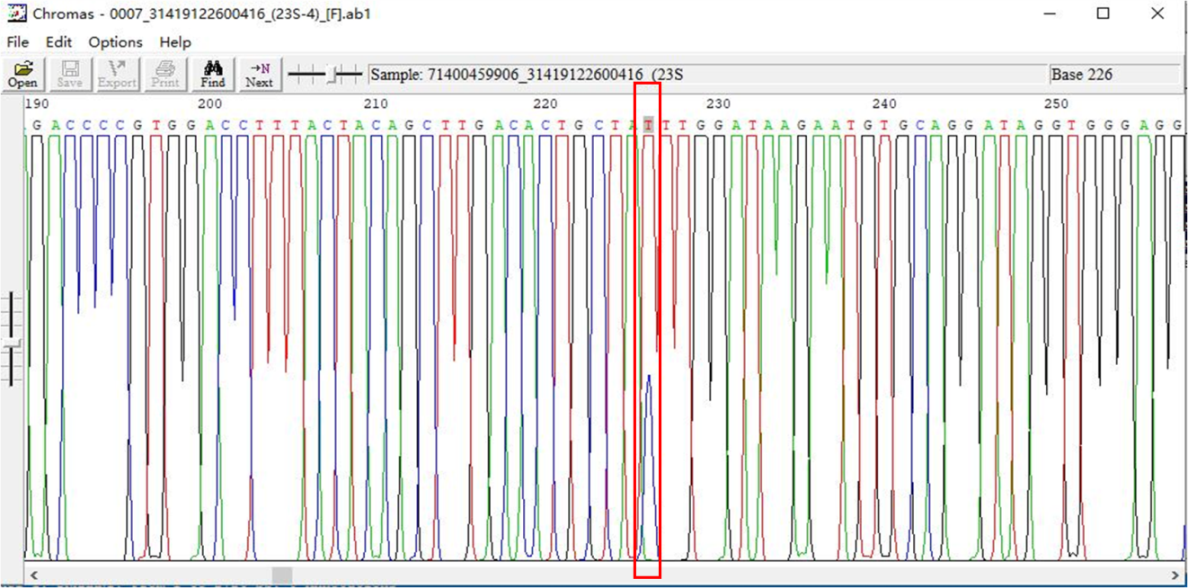


The chromatogram showed a double peak at the position 2113, the peak for “T” being two times higher than the peak for “C”.

1. C2113T mutations present in all of the three copies of the 23S rRNA

GCTCGAAGGTTAATTGATGGGGTTAGCATTAGCGAAGCTCTTGATCGAAGCCCGAGTAAACGGCGGCCGTAACTATAACGGTCCTAAGGTAGCGAAATTCCTTGTCGGTTAAATACCGACCTGCATGAATGGCGTAACGAGATGGGAGCTGTCTCAAAGAGGGATCCAGTGAAATTGTAGTGGAGGTGAAAATTCCTCCTACCCGCGGCAAGACGGAAAGACCCCGTGGACCTTTACTACAGCTTGACACTGCTATTTGGATAAGAATGTGCAGGATAGGTGGGAGGCTTTGAGTATATGACGCCAGTTGTATATGAGCCATTGTTGAGATACCACTCTTTCTTATTTGGGTAGCTAACCAGCTTGAGTTATCCTCAAGTGGGACAATGTCTGGTGGGTAGTTTGACTGGGGCGGTCGCCTCCCAAATAATAACGGAGGCTTACAAAGGTTGGCTCAGAACGGTTGGAAATCGTTCGTAGAGTATAAAGGTATAAGCCAGCTTAACTGCAAGACATACAAGTCAAGCAGAGACGAAAGTCGGTCTTAGTGATCCGGTGGTTCTGTGTGGAAGGGCCATCGCTCAAAGGATAAAAGGTACCCCGGGGATAACAGGCTGATCTCCCCCAAGAGCTCACATCGACGGGGAGGTTTGGCACCTCGATGTCGGCTCATCGCATCCTGGGGCTGGAGCAGGTCCCAAGGGTATGGCTGTTCGCCATTTAAAGCGGTACGCGAGCTGGGTTCAGAACGTCGTGAGACAGTTCGGTCCCTATCTGCCGTGGGCGTAAGAAGATTGAAGAGATTTGACCCTAGTACGAGAGGACCGGGTTGAACAAACCACTGGTGTAGCTGTTGTTCTGCCAAGAGC

1. C2113T mutations present in two of the three copies of the 23S rRNA

GCTCGAAGGTTAATTGATGGGGTTAGCATTAGCGAAGCTCTTGATCGAAGCCCGAGTAAACGGCGGCCGTAACTATAACGGTCCTAAGGTAGCGAAATTCCTTGTCGGTTAAATACCGACCTGCATGAATGGCGTAACGAGATGGGAGCTGTCTCAAAGAGGGATCCAGTGAAATTGTAGTGGAGGTGAAAATTCCTCCTACCCGCGGCAAGACGGAAAGACCCCGTGGACCTTTACTACAGCTTGACACTGCTATTTGGATAAGAATGTGCAGGATAGGTGGGAGGCTTTGAGTATATGACGCCAGTTGTATATGAGCCATTGTTGAGATACCACTCTTTCTTATTTGGGTAGCTAACCAGCTTGAGTTATCCTCAAGTGGGACAATGTCTGGTGGGTAGTTTGACTGGGGCGGTCGCCTCCCAAATAATAACGGAGGCTTACAAAGGTTGGCTCAGAACGGTTGGAAATCGTTCGTAGAGTATAAAGGTATAAGCCAGCTTAACTGCAAGACATACAAGTCAAGCAGAGACGAAAGTCGGTCTTAGTGATCCGGTGGTTCTGTGTGGAAGGGCCATCGCTCAAAGGATAAAAGGTACCCCGGGGATAACAGGCTGATCTCCCCCAAGAGCTCACATCGACGGGGAGGTTTGGCACCTCGATGTCGGCTCATCGCATCCTGGGGCTGGAGCAGGTCCCAAGGGTATGGCTGTTCGCCATTTAAAGCGGTACGCGAGCTGGGTTCAGAACGTCGTGAGACAGTTCGGTCCCTATCTGCCGTGGGCGTAAGAAGATTGAAGAGATTTGACCCTAGTACGAGAGGACCGGGTTGAACAAACCACTGGTGTAGCTGTTGTTCTGCCAAGAGC

1. Wild-type 23S rRNA of *Campylobacter coli*:

23S rRNA *o*f *C. coli* reference strain OR12 (Accession number: CP013733.1).

GCTCGAAGGTTAATTGATGGGGTTAGCATTAGCGAAGCTCTTGATCGAAGCCCGAGTAAACGGCGGCCGTAACTATAACGGTCCTAAGGTAGCGAAATTCCTTGTCGGTTAAATACCGACCTGCATGAATGGCGTAACGAGATGGGAGCTGTCTCAAAGAGGGATCCAGTGAAATTGTAGTGGAGGTGAAAATTCCTCCTACCCGCGGCAAGACGGAAAGACCCCGTGGACCTTTACTACAGCTTGACACTGCTATTTGGATAAGAATGTGCAGGATAGGTGGGAGGCTTTGAGTATATGACGCCAGTTGTATATGAGCCATTGTTGAGATACCACTCTTTCTTATTTGGGTAGCTAACCAGCTTGAGTTATCCTCAAGTGGGACAATGTCTGGTGGGTAGTTTGACTGGGGCGGTCGCCTCCCAAATAATAACGGAGGCTTACAAAGGTTGGCTCAGAACGGTTGGAAATCGTTCGTAGAGTATAAAGGTATAAGCCAGCTTAACTGCAAGACATACAAGTCAAGCAGAGACGAAAGTCGGTCTTAGTGATCCGGTGGTTCTGTGTGGAAGGGCCATCGCTCAAAGGATAAAAGGTACCCCGGGGATAACAGGCTGATCTCCCCCAAGAGCTCACATCGACGGGGAGGTTTGGCACCTCGATGTCGGCTCATCGCATCCTGGGGCTGGAGCAGGTCCCAAGGGTATGGCTGTTCGCCATTTAAAGCGGTACGCGAGCTGGGTTCAGAACGTCGTGAGACAGTTCGGTCCCTATCTGCCGTGGGCGTAAGAAGATTGAAGAGATTTGACCCTAGTACGAGAGGACCGGGTTGAACAAACCACTGGTGTAGCTGTTGTTCTGCCAAGAGC

1. Mutation of 23S rRNA in *C. coli*
2. T2113C mutations present in all of the three copies of the 23S rRNA

GCTCGAAGGTTAATTGATGGGGTTAGCATTAGCGAAGCTCTTGATCGAAGCCCGAGTAAACGGCGGCCGTAACTATAACGGTCCTAAGGTAGCGAAATTCCTTGTCGGTTAAATACCGACCTGCATGAATGGCGTAACGAGATGGGAGCTGTCTCAAAGAGGGATCCAGTGAAATTGTAGTGGAGGTGAAAATTCCTCCTACCCGCGGCAAGACGGAAAGACCCCGTGGACCTTTACTACAGCTTGACACTGCTACTTGGATAAGAATGTGCAGGATAGGTGGGAGGCTTTGAGTATATGACGCCAGTTGTATATGAGCCATTGTTGAGATACCACTCTTTCTTATTTGGGTAGCTAACCAGCTTGAGTTATCCTCAAGTGGGACAATGTCTGGTGGGTAGTTTGACTGGGGCGGTCGCCTCCCAAATAATAACGGAGGCTTACAAAGGTTGGCTCAGAACGGTTGGAAATCGTTCGTAGAGTATAAAGGTATAAGCCAGCTTAACTGCAAGACATACAAGTCAAGCAGAGACGAAAGTCGGTCTTAGTGATCCGGTGGTTCTGTGTGGAAGGGCCATCGCTCAAAGGATAAAAGGTACCCCGGGGATAACAGGCTGATCTCCCCCAAGAGCTCACATCGACGGGGAGGTTTGGCACCTCGATGTCGGCTCATCGCATCCTGGGGCTGGAGCAGGTCCCAAGGGTATGGCTGTTCGCCATTTAAAGCGGTACGCGAGCTGGGTTCAGAACGTCGTGAGACAGTTCGGTCCCTATCTGCCGTGGGCGTAAGAAGATTGAAGAGATTTGACCCTAGTACGAGAGGACCGGGTTGAACAAACCACTGGTGTAGCTGTTGTTCTGCCAAGAGC

1. A2075G mutations present in all of the three copies of the 23S rRNA

GCTCGAAGGTTAATTGATGGGGTTAGCATTAGCGAAGCTCTTGATCGAAGCCCGAGTAAACGGCGGCCGTAACTATAACGGTCCTAAGGTAGCGAAATTCCTTGTCGGTTAAATACCGACCTGCATGAATGGCGTAACGAGATGGGAGCTGTCTCAAAGAGGGATCCAGTGAAATTGTAGTGGAGGTGAAAATTCCTCCTACCCGCGGCAAGACGGAGAGACCCCGTGGACCTTTACTACAGCTTGACACTGCTATTTGGATAAGAATGTGCAGGATAGGTGGGAGGCTTTGAGTATATGACGCCAGTTGTATATGAGCCATTGTTGAGATACCACTCTTTCTTATTTGGGTAGCTAACCAGCTTGAGTTATCCTCAAGTGGGACAATGTCTGGTGGGTAGTTTGACTGGGGCGGTCGCCTCCCAAATAATAACGGAGGCTTACAAAGGTTGGCTCAGAACGGTTGGAAATCGTTCGTAGAGTATAAAGGTATAAGCCAGCTTAACTGCAAGACATACAAGTCAAGCAGAGACGAAAGTCGGTCTTAGTGATCCGGTGGTTCTGTGTGGAAGGGCCATCGCTCAAAGGATAAAAGGTACCCCGGGGATAACAGGCTGATCTCCCCCAAGAGCTCACATCGACGGGGAGGTTTGGCACCTCGATGTCGGCTCATCGCATCCTGGGGCTGGAGCAGGTCCCAAGGGTATGGCTGTTCGCCATTTAAAGCGGTACGCGAGCTGGGTTCAGAACGTCGTGAGACAGTTCGGTCCCTATCTGCCGTGGGCGTAAGAAGATTGAAGAGATTTGACCCTAGTACGAGAGGACCGGGTTGAACAAACCACTGGTGTAGCTGTTGTTCTGCCAAGAGC
